# Combined RNAi targeting human Stat3 and ADAM9 as gene therapy for non-small cell lung cancer

**DOI:** 10.3892/ol.2022.13595

**Published:** 2022-11-14

**Authors:** Liang Chang, Fangchao Gong, Hongfei Cai, Zhihong Li, Youbin Cui

Oncol Lett 11: 1242–1250, 2016; DOI: 10.3892/ol.2015.4018

Following the publication of this paper, it was drawn to the Editors’ attention by a concerned reader that certain of the western blotting assay data shown in Figs. 1C and 4C, and the tumor images shown in Fig. 5A were strikingly similar to data appearing in different form in other articles by different authors. Owing to the fact that the contentious data in the above article had already been published elsewhere, or were already under consideration for publication, prior to its submission to *Oncology Letters*, the Editor has decided that this paper should be retracted from the Journal. The authors were asked for an explanation to account for these concerns, but the Editorial Office did not receive a reply. The Editor apologizes to the readership for any inconvenience caused.

